# Recent advances in transition metal nitrides for hydrogen electrocatalysis in alkaline media: From catalyst design to application

**DOI:** 10.3389/fchem.2022.1073175

**Published:** 2022-12-02

**Authors:** Siyuan Tang, Zhipeng Zhang, Jun Xiang, Xinchun Yang, Xiangqian Shen, Fuzhan Song

**Affiliations:** ^1^ Institute for Advanced Materials, School of Materials Science and Engineering, Jiangsu University, Zhenjiang, China; ^2^ Institute School of Science, Jiangsu University of Science and Technology, Zhenjiang, China; ^3^ Shenzhen Key Laboratory of Energy Materials for Carbon Neutrality, Institute of Technology for Carbon Neutrality/Faculty of Materials Science and Energy Engineering, Shenzhen Institute of Advanced Technology (SIAT), Chinese Academy of Sciences (CAS), Shenzhen, China

**Keywords:** hydrogen oxidation, hydrogen evolution, transition metal nitrides, water electrolyzer, hydrogen fuel cell

## Abstract

Hydrogen (H_2_) has been considered an ideal alternative energy source for solving energy supply security and greenhouse gas reduction. Although platinum group metal (PGM) catalysts have excellent performance in hydrogen electrocatalysis, their scarcity and high cost limit their industrial application. Therefore, it is necessary to develop low-cost and efficient non-PGM catalysts. Transition metal nitrides (TMNs) have attracted much attention because of their excellent catalytic performance in hydrogen electrochemistry, including hydrogen evolution reaction (HER)/hydrogen oxidation reaction (HOR). In this paper, we review and discuss the mechanism of HER/HOR in alkaline media. We compare and evaluate electrocatalytic performance for the HER/HOR TMN catalysts recently reported. Finally, we propose the prospects and research trends in sustainable alkaline hydrogen electrocatalysis.

## Introduction

**FIGURE 1:**
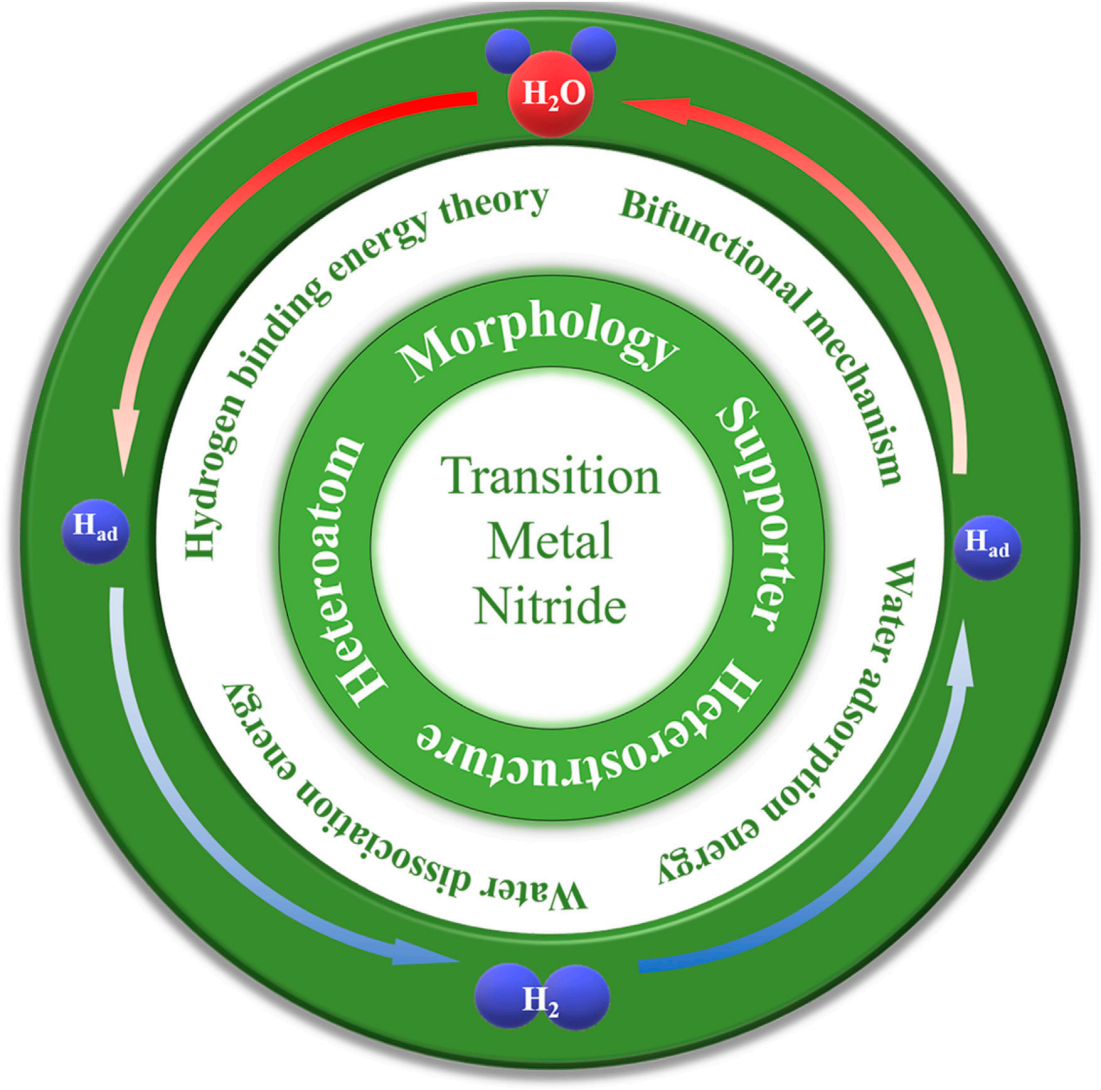
Transition metal nitrides for alkaline electrocatalysis.

Hydrogen (H_2_) is considered an ideal alternative energy source when the earth's fossil fuels will be used up in the next century, owing to its high gravimetric energy density and zero carbon emissions (Jaramillo et al., 2007; Song et al., 2017). Electrocatalytic hydrogen transformation is a proven and convenient strategy for hydrogen economy (Jiao et al., 2015; Cong et al., 2018; Chu et al., 2020). High-purity H_2_ can be produced by hydrogen evolution reaction (HER) and oxygen evolution reaction (OER) in a water electrolyzer with the input of renewable energy resources, while the output of electricity can be achieved by fuel cells through hydrogen oxidation reaction (HOR) (Song et al., 2021). Both electrolyzers and fuel cells can be operated in acidic and alkaline solutions (Ruqia et al., 2018). Due to multiple proton-coupled electron transfer, electrocatalytic hydrogen transformation conversion in alkaline solution faces a large activation barrier, leading to sluggish kinetic activity. Efficient electrocatalysts are therefore required to reduce the energy barrier to achieve an industrially relevant requirement for the hydrogen economy. In the past few years, platinum group metal (PGM)-free catalysts have shown excellent catalytic performance in hydrogen electrochemistry. Among them, transition metal nitrides (TMNs) have attracted much attention due to excellent catalytic performance and robust stability (Figure 1). Bonding with N atoms will change the d-band structure of the transition metal and thus shrink the d-band. In addition, the active centers of N were similar to the electronic structure of Pt, so N can effectively improve the catalytic activity (Han et al., 2018; Yang et al., 2020); this results in thermal stability, chemical stability, and high electronic conductivity. However, although TMNs have developed rapidly in the past decade, they are still far from the requirements of industrial application. Therefore, it is necessary to develop novel TMNs with high activity and stability to achieve a hydrogen economy.

This article discusses the mechanism of HER/HOR in alkaline media. We analyze the catalytic performance and characteristics of the recently reported HER/HOR TMN catalysts before discussing the future development prospects and research trends of TMNs, which will benefit researchers who work in this field.

## Mechanism of hydrogen evolution/oxidation reaction in alkaline solution

### HER mechanism

The elementary steps of HER in alkaline media are as follows:

$$Total:\;2H_{2}O+{2e}^{-}\to H_{2}+{2OH}^{-}$$ (1)

$$Volmer\;Step:M+H_{2}O+e^{-}\to M-H^{*}+{OH}^{-}$$ (2)

$$Hetrovsky\;step:M-H^{*}+H_{2}O+e^{-}\to {\;H}_{2}+M+{OH}^{-}$$ (3)

$$Tafel\;step:\;2M-H^{*}\to H_{2}+2M$$ (4)

M is the active site on the catalyst, and M–H* is the hydrogen adsorption on the active site above the catalyst.

The HER process involves the Volmer, Heyrovsky, and Tafel steps. In the Volmer step, water is dissociated to form a reactive H_ad_ intermediate. In the Heyrovsky step, another H_2_O is adsorbed to M–H^*^ and then reacts with the second electron to form H_2_ and OH^−^. In the Tafel step, two adjacent M–H^*^ are combined to generate H_2_. A whole HER process will follow two pathways—Volmer–Tafel or Volmer–Heyrovsky reactions (Chen et al., 2019)—consisting of three consecutive stages—water dissociation, water adsorption, and hydrogen production.

Generally, the adsorption-free energy of M–H* (ΔG_H*_) is widely accepted as a key description in evaluating the kinetics of the HER catalyst. Optimum ΔG_H*_ to zero is desirable for achieving excellent HER performance, which is regarded as a guide for developing highly efficient HER electrocatalysts. In addition, water adsorption energy ( $E_{ad}^{water}$ ) and the activation energy of water dissociation ( $E_{ac}^{water}$ ) reflect the catalytic behaviors of HER. The lower value of $E_{ad}^{water}$ will result in better affinity between water molecules and the catalyst surface. The decrease of $E_{ac}^{water}$ is proportional to the water dissociation rate because of the smaller the activation energy required. Therefore, computational energies are convenient descriptions when designing high-performance catalysts.

### HOR mechanism

The elementary steps of HOR in alkaline solution are shown as follows:

$$Total:H_{2}+{2OH}^{-}\to 2H_{2}O+{2e}^{-}$$ (5)

$$Tafel\;Step:\;H_{2}+2M\to 2{M-H}^{*}$$ (6)

$$Hetrovsky\;step:H_{2}+M+{OH}^{-}\to {M-H}^{*}+H_{2}O+e^{-}$$ (7)

$$Volmer\;step:\;M-H^{*}+{OH}^{-}\to M+H_{2}O+e^{-}$$ (8)

HOR proceeds in the reverse sequences to HER; in alkaline solution, HOR proceeds through pathways of Tafel–Volmer or Heyrovsky–Volmer. M–H^*^ is formed through the Tafel or Heyrovsky step, and then follows the Volmer step to form H_2_O. In the Tafel step, H_2_ is dissociated in M (active site) without electron transfer to generate M–H^*^ and is adsorbed on the surface of M. During the Heyrovsky step, H_2_ transfers electrons to M on the surface of the catalyst and is dissociated to generate M–H^*^ and H_2_O with the coordination of adjacent OH^−^. Finally, M–H^*^ binds with OH^−^ to form H_2_O in the Volmer step.

Recently, Markovic *et al.* proposed a bifunctional mechanism (Okubo et al., 2019). They took the hydroxyl binding energy as another active descriptor and suggested that OH_ad_ is an important factor for efficient HOR under alkaline conditions. There is much discussion comparing the hydrogen binding energy (HBE) theory and the bifunctional mechanism. Although these two contradictory theories coexist, they succeed in guiding the synthesis of HOR catalysts and reflect the complexity of the HOR mechanisms in alkaline solutions.

## Transition metal nitride for HER/HOR in alkaline solution

In recent decades, transition metal nitrides (TMNs) have exhibited promising HER/HOR performance under alkaline conditions. Therefore, TMNs are gradually applied to energy devices. For example, Liu et al. successfully used 1.5 V single AAA batteries to run the electrolyzer, where bifunctional NiCo nitride/NiCo_2_O_4_/GF could be acted as the anode and cathode (Liu et al., 2019) at the same time. The electrolyzer prepared by Wu et al. only needs 1.644 V to reach a large current of 1A (Wu et al., 2022). Cheng et al. and Hsieh et al. prepared Co-N-C catalyst and reached max power density of 275 and 361 mW cm^−2^ in AEMFCS (Cheng et al., 2022; Hsieh et al., 2022).

The reason why TMNs show outstanding potential is that N can enhance the d-electron density, resulting in contraction of the d-band of TMNs. Such a unique structure will afford TMNs an electronic structure similar to PGM metals, such as Pd and Pt. In addition, the distinguished conductivity and good corrosion resistance make TMNs high-performance. Among various TMNs, Ni_3_N and Co_4_N were synthesized by Shalom et al*.* and Chen et al*.* in 2015 (Chen et al., 2015; Shalom et al., 2015). Subsequently, great efforts have been devoted to optimizing the catalytic activities of TMNs toward HER/HOR electrocatalysts. Different design strategies were used to improve the stability of various TMN catalysts. For example, Yan et al*.* (Yan et al., 2021) ensured high structural stability by making TMNs contact closely with MXene and forming a strong interface junction. Yuan et al. (2020) significantly inhibited the oxidation of Co_4_N through a carbon shell to improve stability. Therefore, while improving catalytic performance, some design strategies can also improve the stability of TMNs.

In this section, the strategies for the rational design of TMN electrocatalysts will be classified and summarized; the outstanding TMN-based electrocatalysts are compared in Table 1.

**TABLE 1:** Transition metal catalysts for the alkaline HER/HOR.

Catalyst	Electrolyte	HER			Reference
		η_10_ (mV)	η_100_ (mV)	Tafel slope (mV/dec)	
Ni_3_N/NF	1.0M KOH	121	254	-	Xing et al. (2016)
CoN@CC	1.0M KOH	97	-	93.9	Xue et al. (2018)
Cu_3_N	1.0M KOH	118	280	122	Panda et al. (2019)
Fe_2_N/CC	1.0M KOH	110	125	41.2	Wu et al. (2020)
FeNi_3_N	1.0M KOH	75	210	98	Zhang et al. (2016)
Ni_3_N–CeO_2_/TM	1.0M KOH	80	-	122	Sun et al. (2018)
Cr-Ni_3_N/Ni	1.0M KOH	37	110	47	Wu et al. (2021)
Co_3_O_4_–Co_4_N	1.0M KOH	90	-	57.8	Liu et al. (2019)
Ni_3_N-VN	1.0M KOH	64	218	37	Yan et al. (2019)
Ni_3_N-V_2_O_3_	1.0M KOH	57	-	50	Zhou et al. (2021)
NiMoN	1.0M KOH	109	161	95	Zhang et al. (2016)
NiMoN/Ni_3_N	1.0M KOH	28	93	49	Hua et al. (2021)

Catalyst	Electrolyte	HOR		Reference
		Current density (mA/cm^2^)	Mass loading (mg/cm^2^)	
		@ 50 mV		
Ni/SC	0.1 M KOH	1.2	0.30	Yang et al. (2019)
Ni/N-CNT	0.1 M KOH	1.3	0.25	Zhuang et al. (2016)
np−Ni_3_N	0.1 M KOH	1.7	0.16	Pei et al. (2019)
Ni_4_Mo	0.1 M KOH	2.6	0.2	Wang et al. (2021)
Ni_3_N	0.1 M KOH	2.2	0.32	Wang et al. (2019)

### Synergistic metal-support interactive TMN catalysts

Synergistic metal-support interaction (SMSI) can be observed between metal catalysts and solid supports (Song et al., 2015; Song et al., 2020). SMSI provides possibilities for designing catalysts, modulating their d-band of catalytic sites and optimizing their binding energy of intermediates, thus resulting in accelerated reaction kinetics. Indeed, solid support is important for determining SMSI (Song et al., 2015; Song et al., 2018). An ideal solid support not only improves SMSI and re-modifies the electronic structure of metal sites but also restrains the agglomeration of metal species.

Carbon-based materials are excellent solid supports due to their large surface area, tailorable porous structure, high resistance, and convenient surface functionalization. Vulcan-XC 72R, a commercial carbon, possesses suitable surface area, pore size, and low-cost. Ni_3_N nanoparticles can be well-dispersed on a Vulcan-XC 72R surface and exhibit a robust HOR performance (Ni et al., 2019). In addition, a series of novel carbon-based materials have been developed. Yuan et al*.* (2020) designed cobalt nitrides embedded in nitrogen-doped carbon (Co_4_N@NC). NC acted as a conductive network to supply abundant active sites and efficiently retained the surface oxidation of Co_4_N. SMSI between Co_4_N and NC optimized DOS near the Fermi level, resulting in an enhancement of HER kinetics. Moreover, such improved HER performance has been observed in CoN/N-doped carbon nanotubes because one-dimensional nanotubes can provide ordered channels for rapid mass-diffusion and electron transfer. Porous metal–organic frameworks (MOFs) have emerged as precursors to obtaining TMN nitrides under controlled nitridation. Feng et al*.* (2019) synthesized MOF-74-derived NiCoN catalysts with porous rodlike structures. The abundant synergistic active sites obtained have accelerated electron transfer and modulated the chemical environment, resulting in all-pH HER performance (Feng et al., 2019). A series of nickel nitrides and cobalt nitrides derived from MOFs were reported (Hu et al., 2020; Wang et al., 2022). Jia et al*.* (2022) reported the construction of Ni_3_N/MOF-74, which shows a remarkably HER performance with an overpotential of 73 mV to afford 10 mA cm^−2^ in a 1.0 M KOH solution (Jia et al., 2022).

Transition metal carbides/nitrides (MXenes) as new two-dimensional materials are considered promising candidates for solid supports (Zhang et al., 2021). MXenes discovered by Gogotsi and colleagues in 2011 possess a larger specific surface area, high conductivity, good hydrophilicity, robust stability, and various functional groups (Naguib et al., 2011). Very recently, the effect of MXenes’ support on HER performance has been explored. CoN_x_/MXenes have been reported by Li et al*.* (Li et al., 2021): the construction of CoN_x_ and MXenes can expose rich active sites, optimize electronic structure, and promote intermediate adsorption and desorption, thus improving HER kinetics. The DFT results prove furthermore that more electrons accumulate at the surface of the Co atom, arising from the strong charge transfer caused by the interface coupling between CoN_x_ and MXenes. These improve mass/charge transport and promote water adsorption/activation in the Volmer step in HER. These studies have proven that MXenes are a promising support for exploring and developing cost-effective catalysts with high activities.

### Hetero-interfacial TMN catalysts

Hetero-interface engineering is another promising strategy for developing high-performance PGM-free electrocatalysts. The interface effects will reconstruct active sites by modifying the electrochemical environment between two different active components. The reconstructed active sites at the heterogeneous interfaces can show higher activity than individual components. Up to now, the modifications of TMN-based interface catalysts, such as transition metal nitrides/transition metal (TMNs/TM), transition metal nitrides/transition metal nitrides (TMNs/TMNs), and transition metal nitrides/metal oxides (TMNs/MOs), have been reported.

It is well-known that the construction of TMNs/TM can efficiently improve conductivity and optimize the binding behaviors of catalytic sites toward reactants, intermediates, and products. In 2018, Ni_3_N/Ni interfacial catalysts were synthesized by electrodeposition combined with controlled nitridation, which is a pioneering report on a Ni_3_N-based electrocatalyst with bifunctional HER/HOR activity (Song et al., 2018). The construction of a Ni_3_N/Ni heterogeneous interface benefited the electron transfer at the interfacial sites and improved electrophilic properties. Strong electron redistribution occurred at the interface between Ni_3_N and Ni, which significantly promoted initial water adsorption and reduced the energy barrier of the subsequent water dissociation. Moreover, the H* species were achieved at the interfacial region. The ΔG_H*_ of the interfacial site was as low as 0.01 eV, close to the ideal value of zero—resulting in an exceptional HER/HOR catalytic property. This group further developed a Co_2_N/Co interface with better robustness and Co tolerance (Song et al., 2019). Other TMNs/TM systems have been explored by Fan group (Fan et al., 2018) and Sun group (Sun et al., 2020). The former designed an atomic in-growth Co–Ni_3_N interface (Zhu et al., 2018). The epitaxial Co–Ni_3_N interfaces in nanoscale are responsible for robust stability due to small interfacial energy. In addition, the nanoconfinement effect can accelerate the electron transfer between Co and Ni_3_N domains at the epitaxial interface, resulting in excellent electrocatalyzing hydrogen production. Wang group further explored the chemical anchoring and electronic regulation at Ni_3_N/Ni. The formation of the Ni−N bond made the heterogeneous interface produce unique electronic states by changing the electrons’ state. Strong electron redistribution occurred at the interface between Ni_3_N and Ni, where the water formation reaction barrier could be significantly reduced, resulting in enhanced HOR performance (Wang et al., 2021).

The TMNs/TMN heterogeneous interface has attracted much attention due to its faster charge transfer and compatibility. CoN/Ni_3_N catalysts with a grass-like structure were synthesized *via* a controlled pyrolyzation (Ray et al., 2018). This generated abundant active centers with expeditious charge and mass transportation, inducing an enhanced HER property in 1.0 M KOH solution. A similar phenomenon was observed in Ni_3_N/Co_3_N (Huang et al., 2021) and Ni_3_N/Mo_2_N (Dai et al., 2022). In 2022, Song et al*.* reported a novel Ni_3_N/Co_2_N epitaxial interface, which exhibited superior bifunctional HER/HOR performance with respect to the state-of-the-art Pt catalyst due to an accelerating electron transfer at interface region. The bifunctional Ni_3_N/Co_2_N electrocatalysts exhibited excellent performance in a Swagelok type Ni-H_2_ battery with robust stability, demonstrating excellent rechargeability over 5000 cycles (Song et al., 2022).

Heterogeneous TMNs/MOs have proven to be efficient interfacial catalysts. Although MOs are not active for hydrogen electrochemistry, the addition of MOs benefits the modulation of the d-band and electronic structure of MOs, accelerating the kinetics of hydrogen electrochemistry. Sun et al*.* reported a Co_4_N/CeO_2_ interfacial system, where the *in situ* growth of CeO_2_ can efficiently modify the electronic structure of the nearby Co_4_N surface, favoring H_2_O dissociation and intermediated H^*^ adsorption (Sun et al., 2020). Zhou group prepared Ni_3_N/V_2_O_3_ by nitriding a NiV–LDH (layered double hydroxide) precursor (Zhou et al., 2021). The introduction of V_2_O_3_ made the interface couple between Ni_3_N and V_2_O_3_; the interfacial interaction resulted in charge redistribution and generated more unoccupied electrons on the N and O in the interface region, which efficiently optimized the H_2_O adsorption/desorption ability. This hypothesis was further confirmed by DFT calculation, where Ni_3_N/V_2_O_3_ exhibits lower $E_{ad}^{water}$ , $E_{ac}^{water}$ , and ΔG_H*_ than Ni_3_N.

Other TMN-based interface catalysts have also been reported. Sun et al*.* (2018) explored the abundant defects on a CoN/Ni_2_P surface (Sun et al., 2018). Hu et al*.* (2019)developed a series of CoN/Co_2_P-based interfacial catalytic sites (Guo et al., 2018; Hu et al., 2019). Ren et al designed Ni_3_N/Ni(OH)_2_ with excellent HER/HOR performance (Ren et al., 2022). The introduction of nickel hydroxide reduced the center of the Ni d-band center and thus reduced the adsorption strength. The strong electronic coupling made Ni_3_N negatively charged by interface engineering with seamless heterojunctions. The negatively charged Ni sites improved the bond interaction between the Ni sites and surface H* groups. Ni_3_N/Ni(OH)_2_ surface engineering promoted water dissociation and hydroxyl adsorption in the Volmer step toward HER and HOR.

As shown previously, interface engineering has become an effective strategy for improving catalyst activity by generating electron redistribution at the interface and realizing synergistic effects.

### Heteroatom doping TMN catalysts

Heteroatom doping can be utilized to modify the charge redistribution of host materials. In contrast to interfacial engineering, heteroatom doping can retain the original structure and morphology of host materials. Due to different electronegativities, heteroatoms adjust the electronic structures of host catalytic sites and gear up the catalytic performance of electrocatalysts, killing “two birds with one stone” (Zhang et al., 2021).

Recently, Zhu group prepared Co doping for a Ni_3_N heterostructure catalyst (Zhu et al., 2018). The XRD results confirmed that the Ni_3_N phase did not change with the addition of Co. Epitaxial in-growth Co promotes electrons transferal from Co to Ni_3_N, where these added electrons can stabilize the H atom, thus reducing ΔG_H*_ to improve HER performance. Ni doping of CoN is further demonstrated by Yu et al*.* (Yu et al., 2019). The enhanced performance was mainly due to CoN catalytic sites being modified by nearby Ni, leading to more active sites, improved conductivity, and fast charge transfer. In addition, Zhang et *al.* doped V into Ni_3_N/Ni to fabricate V doping Ni_3_N/Ni (Zhang et al., 2021). The charge redistribution is mainly confined to the N and Ni sites nearest to the doping V. The coupling of Ni and V dopant can promote water adsorption and optimize the value of ΔG_H*_. Zhang et *al.* reported a V-doped Ni_3_N nanosheet, denoted as V doping Ni_3_N NS(Zhang et al., 2021). V doping can effectively adjust the electronic structure of Ni_3_N, reduce the energy barrier of RDS, and promote dehydrogenation kinetics and H* adsorption/desorption. Wu et al*.* further proved that Cr doping can downshift the high unoccupied d-orbital of Ni_3_N (Wu et al., 2021). Cr doping exists as a Cr-n_6_ state, which can redistribute the surface electronic state of Ni_3_N to reduce the average d-band energy of Ni_3_N. The downshift of d-band energy can strengthen the orbital coupling between the unpaired electrons of O 2p and the unoccupied states of Ni 3d—beneficial to the adsorption and dissociation of water. Cr doping Ni_3_N can also promote the subsequent solution of H through the synergistic effect. Therefore, the doping of Cr enhanced the electron coupling with water molecules and promoted the dissociation kinetics of water molecules, thereby improving the HER performance, which is very close to the performance of Pt/C.

Heteroatom dopant can adjust electron density, modulate the orbit of catalytic sites, improve the conductivity of host materials, and retain the intrinsic component and structure of the host catalyst, thus improving the catalytic performance of TMNs. These results unambiguously show the advantage of the heteroatom dopant in improving catalytic performances.

## Conclusions and perspectives

Although significant progress has been made in hydrogen electrochemistry, most of the reported HOR/HER catalysts in the laboratory still cannot meet the demands of industrial devices, especially PGM-free electrocatalysts. Herein, we envision the challenges and opportunities in this field:

1) Although DFT calculation is very helpful for identifying the factors affecting catalyst performance, the theoretical models cannot easily describe the real catalytic conditions, especially the thermodynamics of gas reactions, which may lead to deviation from the ideal liquid conditions under the applied potential. Thus, more accurate theories are needed to explain the complex reaction process and responsibility for developing novel PGM-free electrocatalysts.
2) Compared to PGM electrocatalysts, TMNs possess moderated chemical stability in hash conditions. Although many TMNs have achieved excellent catalytic performance, long-term stability at high current is the main challenge of transition metal nitrides for HER. Moreover, the performance of TMNs for alkaline HOR in the laboratory is far from practical in application. In addition, because industrial H_2_ supplies are mainly provided by steam reforming, excellent CO tolerance performance is highly desirable for HOR electrocatalysts. However, up to now, only a few electrocatalysts have exhibited moderated CO tolerance.
3) At present, most PGM-free HOR catalysts are evaluated in three electrode systems. However, the performance of TMN catalysts in hydrogen fuel cells is generally moderated, owing to the oxidation of active metal sites, which results in a rapid decrease in catalytic performance. In addition, the Hash-operated condition of water electrolysis and fuel cells will result in the changed surface oxidation of TMN electrocatalysts. Therefore, in the future, attention should be paid to improving the stability and antioxidants of PGM-free electrocatalysts under harsh reaction environments.

In summary, the present review introduces the basic and related theories of HER/HOR and analyzes the innovative HER/HOR catalysts reported recently. The different design strategies for adjusting the electronic configuration to optimize the thermodynamic process of hydrogen adsorption/desorption of the catalyst were compared: 1) solid support synergism; 2) hetero-interface engineering; 3) heteroatom doping. The research on catalysts based on TMNs is still very limited, and there is still much room for improvement, especially using HOR of TMNs. We believe that, with continuous research in this area, the commercial application of hydrogen energy will be greatly promoted.
